# High aspect ratio graphene oxide: a highly efficient plasmid DNA deliverer for plant seed

**DOI:** 10.3389/fpls.2026.1855385

**Published:** 2026-06-11

**Authors:** Yue Pan, Xinyu Li, Zhongzhu Yang, Guangzhi Ma, Cheng Jiang

**Affiliations:** 1 Institute of Thoracic Oncology, West China Hospital of Sichuan University, Chengdu, China; 2 Chengdu Institute of Biology, Chinese Academy of Sciences, Chengdu, China; 3 University of Chinese Academy of Sciences, Beijing, China; 4 School of Pharmacy, Chengdu University of Traditional Chinese Medicine, Chengdu, China

**Keywords:** graphene oxide, high aspect ratio, plant genetic engineering, plasmid DNA delivery, tissue culture-free

## Abstract

**Introduction:**

Plant genetic engineering plays a central role in crop improvement and the biosynthesis of natural products. However, the plant cell wall, as a natural barrier, restricts the effective delivery of exogenous biomolecules, especially the delivery of plasmid DNA into the interior of plant cells.

**Methods:**

Here, we introduce a novel form of graphene oxide characterized by a high aspect ratio, synthesized through low-voltage, low-current, and prolonged electrochemical oxidation in a 0.5 mol/L NaOH aqueous solution. To evaluate the efficacy of HARGO in delivering exogenous genes, the *Poa crymophila* Keng seeds were immersed and germinated in a solution containing HARGO and plasmid DNA pEG100-PcNAC2-EGFP.

**Results:**

The high aspect ratio graphene oxide (HARGO) exhibits advantageous characteristics, including a small lateral dimension and single-atom layer thickness (0.414 nm), which can directly penetrate plant tissues and even cell walls. Notably, HARGO can physically adsorb plasmid DNA and facilitate its entry into plant cells for gene delivery without requiring chemical modifications such as polyethylene glycol (PEG) or polyethyleneimine (PEI). Fluorescence signals corresponding to EGFP were successfully observed in the emerged seedlings, with a positive transformation rate reaching 90%.

**Discussion:**

These findings offer a novel tissue culture-free plasmid DNA delivery tool for plant genetic engineering, which will significantly impact the advancement of plant biotechnology.

## Introduction

Plant biotechnology plays a crucial role in addressing global food security and energy demands, as well as having significant applications in the field of biopharmaceuticals (Etit et al., 2024; Reid et al., 2020; Tyczewska et al., 2023). In agriculture, genetic improvement techniques can develop varieties that are high-yielding, high-quality, resistant to herbicides, diseases, and pests, as well as tolerant to abiotic stresses (Liu et al., 2021). In the medical field, genetically engineered plants can produce valuable microbial drugs and recombinant proteins, while bioengineered plants can also generate cleaner and more efficient biofuels (Desai et al., 2010; Eidenberger et al., 2023; Pradhan and Mbohwa, 2014).

Although plant biotechnology has made numerous advancements over the past few decades, the genetic transformation of many plant species remains highly challenging (Su et al., 2023). One of the primary challenges is to penetrate the tough and multi-layered plant cell walls to effectively deliver biomolecules into the interior of the cells (Squire et al., 2023; Su et al., 2023). Currently, only a limited number of delivery tools are employed to achieve this goal, each with its own constraints. *Agrobacterium*-mediated transformation remains the predominant approach for plant genetic transformation, yet its efficiency is constrained by host range bias (favoring dicots over monocots), tissue specificity (requiring actively proliferating cells), and strong genotype dependence (Azizi-Dargahlou and Pouresmaeil, 2024). Biolistic transformation (gene gun) is another widely used tool for plant genetic engineering, enabling DNA/RNA delivery to diverse species including monocots and Agrobacterium-resistant varieties. Nevertheless, it is limited by localized transformation at bombardment sites, tissue damage from high-speed particles, constraints on sample size and positioning, high DNA and microcarrier (gold/tungsten) costs, and frequent multi-copy insertions with DNA fragmentation that cause gene silencing and genomic instability (Lv et al., 2020). For the transient expression of foreign proteins in plants, technologies based on the tobacco mosaic virus, such as Geneware, and other plant viral vectors like the potato X virus and cowpea mosaic virus, facilitate the industrial-scale production of relevant proteins. However, these viral vectors have limited compatibility with specific plant species and the size of the expression vector, which restricts the choice of host plants and hinders the simultaneous expression of large proteins or multiple proteins. Furthermore, the use of viral vectors for transient expression in gene editing systems is typically subject to stringent regulatory scrutiny due to their pathogenic origins and the potential for some viruses to integrate their genetic material into the plant genome (Abrahamian et al., 2020; Chung et al., 2006; Zaidi and Mansoor, 2017). Therefore, to address these challenges, researchers are actively exploring new technologies and methods to enhance the efficiency and applicability of plant genetic transformation.

The application of nanomaterials for gene delivery to animal cells has been extensively researched; however, their potential in plant systems remains underexplored. While research indicates that plant cells can absorb nanomaterials, many of these investigations focus primarily on the delivery of non-functional payloads or depend on mechanical methods—such as gene guns or ultrasound—to promote the penetration of nanoparticles through the plant cell wall (Yan et al., 2022). Recently, several nanomaterials, such as mesoporous silica nanoparticles (MSNs), DNA nanostructures, silicon carbide whiskers (SCWs), layered double hydroxide (LDH) clay nanosheets, and carbon nanotubes (CNTs), have shown the potential to penetrate the plant cell wall and deliver functional biological payloads without the need for strong mechanical assistance. In the study of MSNs, MSNs have been shown to efficiently deliver plasmid DNA to *Arabidopsis* roots and deliver siRNA to leaves of mature plants through passive transport (Cai et al., 2024; Chang et al., 2013). SCWs have enabled the delivery of genes to undifferentiated plant tissues and explants in suspension by co-culturing and vertexing with plant cells and DNA, promoting the stable transformation and screening of transgenic plants. It is speculated that SCWs may allow DNA entry into cells by penetrating or tearing the cell wall, but this mechanism is not suitable for subcellular/tissue targeting or testing in whole plants, and may affect transformation efficiency and cell health (Matsushita et al., 1999; Su et al., 2023). LDH clay nanosheets have effectively delivered RNAi molecules to tobacco, achieving gene silencing and providing a new direction for the development of plant bio-nanotechnology. Although LDH clay nanosheets have not yet been used for plasmid DNA delivery, their potential is promising (Mitter et al., 2017). CNTs, emerging as carriers for plant plasmid DNA delivery, have demonstrated the ability to effectively adsorb and deliver plasmid DNA to plant cells, resulting in the expression of fluorescent proteins. However, the size of chemically modified CNTs increases significantly after binding to plasmid DNA, restricting their entry to either plant protoplasts or the surface cells of plant tissue (Demirer et al., 2019; Dunbar et al., 2022; Pawar et al., 2023). In summary, the prospects for the application of nanomaterials in plant gene delivery are extensive; however, challenges remain, including tissue penetration, cell health, and subcellular targeting, necessitating further research and optimization.

Graphene oxide (GO) is an oxidized form of graphene characterized by a substantial presence of oxygen-containing functional groups, mainly including carboxyl, carbonyl, and epoxy groups. GO exhibits properties such as a large surface area, strong hydrophilicity, and low biological toxicity (Zhang et al., 2021). GO is typically synthesized from graphite through strong acid oxidation employing methods such as Brodie’s method, Staudenmaier’s method, or Hummers’ method (Korucu et al., 2023; Peng et al., 2015). Existing research indicates that graphene oxide prepared by traditional methods may enter plant cells through passive transport or spontaneous membrane penetration. Moreover, under specific ionic conditions, GO can also bind to double-stranded DNA or RNA (Dong et al., 2022; Li et al., 2022). Currently, to utilize graphene oxide as a vector for delivering genetic material (Plasmid/DNA/RNA) into plant cells, chemical modification with polyethylene glycol (PEG) and polyethylenimine (PEI) is typically required (Feng et al., 2013). Following chemical functionalization, graphene oxide forms stable, small-sized nanocomplexes upon genetic material adsorption, thereby effectively penetrating the plant cell wall to enable delivery into intact plant cells (Desai et al., 2010; Li et al., 2013). To date, there have been no reports of plasmid DNA delivery into plant cells using chemically unmodified GO.

In this study, a new type of high aspect ratio graphene oxide (HARGO) material was developed through electrochemical oxidation at low pressure, low current and for a long time in dilute sodium hydroxide aqueous solution. HARGO can physically adsorb plasmid DNA in aqueous solution and stably carry plasmid DNA through the rigid plant cell wall and into the seed embryo part to cause foreign gene expression. This innovative approach offers new opportunities to enhance the efficiency and applicability of plant genetic transformation.

## Materials and methods

### Plant materials

The wheat variety is spring wheat Zhongkemai 138 (ZKM138), and the seeds were provided by Professor Tao Wang of the Chengdu Institute of Biology, Chinese Academy of Sciences.

The variety of *Poa crymophila* Keng is *Poa crymophila* Keng cv. Qinghai (The seed was provided by Professor Xinrong Ma of the Chengdu Institute of Biology, Chinese Academy of Sciences).

### Preparation of HARGO

Graphite rods with a 5 mm diameter (spectral purity, model YL-BDC-100) were used as the anode, while a Pt electrode of 99.99% purity (10*10*0.1 mm) served as the cathode. In a 400 mL solution of 0.5 mol/L NaOH, the electrode spacing was set at 6.5 cm, with both electrodes submerged to a depth of 6.5 cm in the electrolyte. Electrochemical oxidation was carried out at a voltage of 3.5 V and a current of 0.03 A for 96 h. The resulting brownish-yellow solution after electrolysis was dialyzed against deionized water using MD44–3500 dialysis tubing until the pH stabilized at 6.8. The solution was then concentrated at a constant temperature of 40°C in an oven after dialysis. The HARGO concentration was determined by drying 100 mL of the concentrated solution to a constant weight.

### Apparatus for characterizations

The Zeta potential was measured by Nano9200 nanoparticle analyzer (Haixinrui). Atomic force microscopy (AFM) images of the samples were captured in tapping mode using a Nano Scope IIIA AFM, with samples prepared by spin coating a diluted aqueous solution onto a mica substrate at 1000 r.p.m. Fourier transform infrared spectroscopy (FT-IR) spectra were recorded on a PE Spectrum 100 spectrometer, utilizing either a film or KBr discs. Ultraviolet-visible spectroscopy (UV-Vis) spectra were obtained using a PerkinElmer Lambda 35 UV/Vis Spectrometer for analysis in the ultraviolet-visible range.

### Titration test

For the HARGO group, 10 mg of HARGO was dissolved in 25 mL of 0.1 mol/L HCl aqueous solution (adjusted to pH = 1.08), and then titrated with 0.1 mol/L NaOH aqueous solution. For the control group, 25 mL of 0.1 mol/L HCl aqueous solution (adjusted to pH = 1.08) was taken and titrated with 0.1 mol/L NaOH aqueous solution. During the titration process, the pH value change of the solution was detected in real time by a pH meter (PHS-3E, Shanghai, China). The titration curve was plotted by Origin v2021 software.

### Construction of plasmid DNA pEG100-PcNAC2-EGFP

Took 20 µL of plasmid DNA backbone pEG100-EGFP with a concentration of 100 ng/µL. Then, perform enzymatic digestion with BamH I and BbvC I according to the instructions. Subsequently, performed agarose gel electrophoresis and gel recovery to obtain the digested pEG100-EGFP. Used the Plant Total RNA Extraction Kit (BL1180A) to extract the total RNA of *Poa crymophila* Keng, and then detect the concentration and quality of the extracted total RNA by agarose gel electrophoresis. Used the reverse transcription kit (Cat. No. 218161) to synthesize cDNA using RNA as a template. Performed PCR amplification with specific primers and sequencing to obtain a *PcNAC2*-specific fragment with one intron deletion. The upstream sequence of the specific primer is 5’-GAATTCATGGGGATGGCCGTGCGCAGG-3’, and the downstream sequence is 5’-CCTCAGCGGAGTCGCTCAAGAAGGGAGCCGGCATGCC-3’. Then, performed PCR amplification on this specific fragment using primers with restriction endonuclease-specific sequences of BamH I and BbvC I added at both ends, and performed agarose gel electrophoresis and gel recovery to obtain the target DNA fragment. Add the obtained target DNA fragment aqueous solution (volume 20 µL, concentration 100 ng/µL) to restriction endonucleases BamH I and BbvC I for enzymatic digestion. Then, performed agarose gel electrophoresis and gel recovery again to obtain the digested target DNA fragment. Use the T4 DNA ligase kit (Cat. No. M02002) to ligate the digested plasmid DNA pEG100-EGFP and the target DNA fragment to obtain plasmid DNA, specifically pEG100-PcNAC2-EGFP. The length of the plasmid DNA is 11, 798 bp.

### Adsorption of plasmid DNA onto HARGO

The constructed pEG100-PcNAC2-EGFP was transformed into DH5α *Escherichia coli* and cultured on LB solid medium containing kanamycin. After inverted culture at 37 °C for 2 days, a single colony was picked from the plate and inoculated into liquid medium containing kanamycin, and cultured overnight with shaking at 200 rpm at 37 °C to make the bacteria grow to the late logarithmic growth phase. The cultured bacterial solution was transferred to a centrifuge tube. After centrifugation at 4000 rpm for 10 minutes, the supernatant was discarded and the bacterial cell pellet was collected. Subsequently, plasmid DNA was extracted according to the instructions of QIAGEN Plasmid Maxi Kit (Cat. No. 12163), and the extracted plasmid DNA was quality tested and quantified by NanoDrop 2000/2000c (NanoDrop Technologies; Thermo Fisher Scientific, Inc., Pittsburgh, PA, USA).

A mixture was prepared by combining a 0.5 mg/mL aqueous solution of HARGO with a 110 ng/µL aqueous solution of plasmid DNA (pEG100-PcNAC2-EGFP, 11, 798 bp in length) in equal volumes. The components were gently agitated at room temperature to ensure thorough mixing, yielding an aqueous solution of HARGO with adsorbed plasmid DNA.

### Delivery of plasmid DNA to plant seeds via co-cultivation with HARGO

In 2.0-mL polyethylene (PE) tubes, a small amount of dry substrate soil was placed, *Poa crymophila* Keng seeds were sown, and covered with a thin layer of dry substrate. The soil was then irrigated with the aqueous solution of HARGO adsorbed with plasmid DNA, ensuring that the soil was moist with the solution level about 1 mm above the soil surface. The tubes were kept in darkness at room temperature for two days to facilitate the delivery of plasmid DNA to the seeds by HARGO. Following the dark treatment, the plants were transferred to a phytotron with a photoperiod of 12 hours light/12 hours darkness, a light intensity of 300 photosynthetic photon flux density (PPFD), a temperature of 22-25°C during the light hours, and 18-20°C during the dark hours.

### Laser confocal microscopy detection

Leaf sections from 14-day-old seedlings were obtained, and 100 µm thick transverse sections were cut using a cryostat (Leica CM1950). These sections were promptly examined under a laser confocal microscope (Leica TCS SP8), using 488-nanometer excitation light and a detection wavelength range of 500–540 nanometers.

### PCR analysis

Stems and leaves of *Poa crymophila* Keng from 14-day-old seedlings were sampled and processed for PCR amplification following the instructions provided in the Plant Leaf Direct PCR Kit (Cat. No: TP-0211T). The PCR reaction mixture consisted of 20 µL, comprised of 10 µL of 2×Leaf PCR Easy™ Mix, 1 µL each of upstream and downstream primer (10 mM), 1 µL of sample lysate, and 7 µL of ddH_2_O. The upstream primer sequence was 5’-ATGGGGATGGCCGTGCGCAGG-3’, and the downstream primer sequence was 5’-CTCAAGAAGGGAGCCGGCATGCC-3’. The PCR program included initial denaturation at 95°C for 5 min, followed by 32 cycles of denaturation at 95°C for 40 s, annealing at 67°C for 40 s, extension at 72°C for 1 min, and a final extension at 72°C for 5 min. After PCR, 2 µL of 10×DNA loading buffer was added for agarose gel electrophoresis detection.

### Data compilation and statistical analysis

Data were organized using Microsoft Excel 2023. Statistical analyses were conducted using IBM SPSS v24.0 (SPSS, Chicago, USA).

## Results

### Preparation and characterization of HARGO

**Figure 1:**
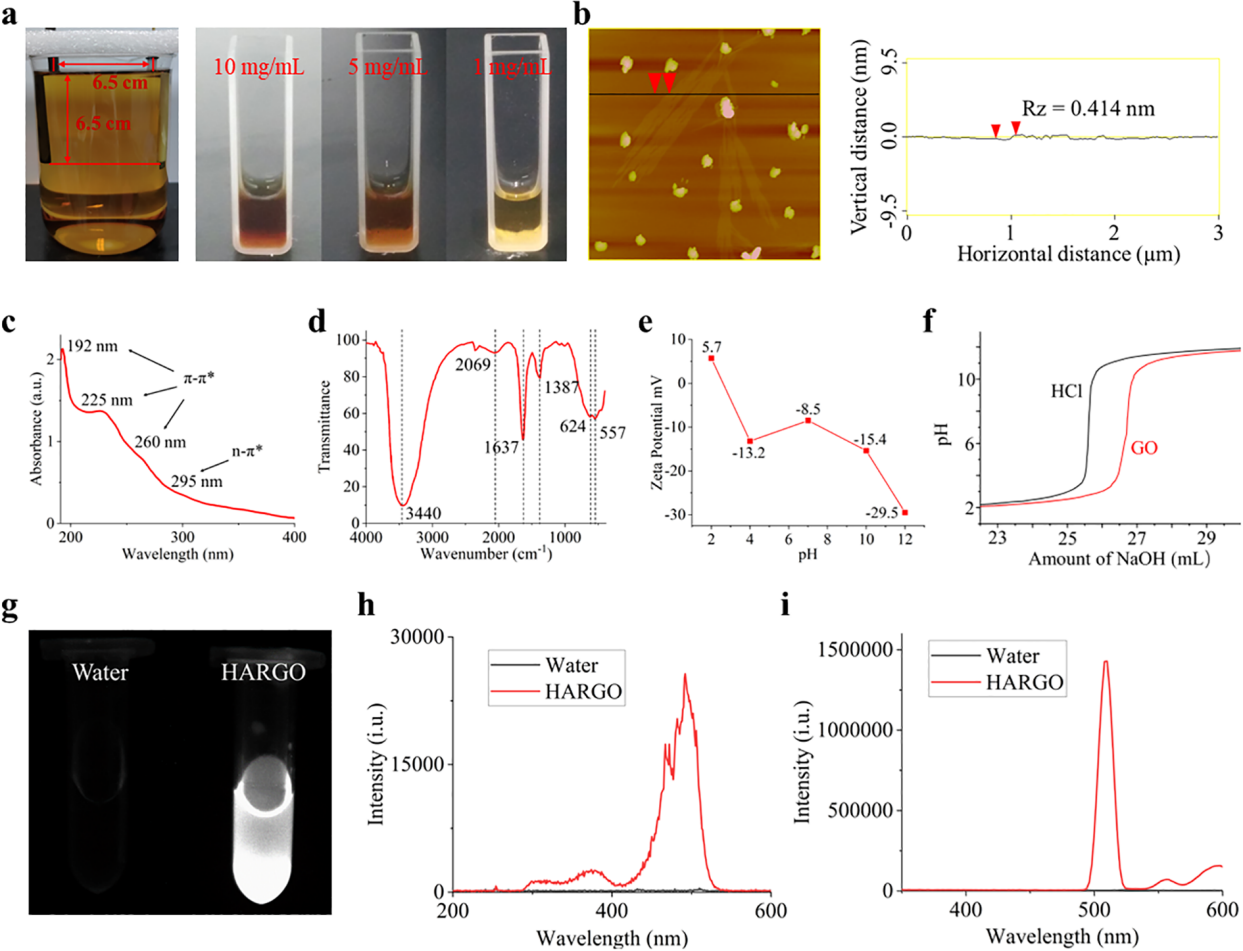
Preparation and characterization of high aspect ratio graphene oxide (HARGO). **(a)**, The left image displays a digital photograph taken after 96 hours of electrolysis, in which the anode is a graphite rod and the cathode is a Pt electrode, positioned 6.5 cm apart and submerged 6.5 cm below the liquid surface. The right image illustrates HARGO aqueous solutions at concentrations of 10, 5, and 1 mg/mL, respectively. **(b)**, The left image presents an atomic force microscope (AFM) image of HARGO, with the point-like substance identified as NaOH impurity. The right image depicts the height profile. **(c)**, UV-vis Spectrum of HARGO at a concentration of 0.5 mg/mL. **(d)**, Fourier transform infrared spectrum of HARGO. **(e)**, Zeta Potential Analysis of HARGO at a concentration of 0.1 mg/mL. **(f)**, Titration curve of HARGO. **(g)**, Digital photograph of fluorescence emitted by 1 mg/mL HARGO aqueous solution under excitation light with a wavelength of 350 nm. **(h)**, Excitation spectrum of HARGO. **(i)**, Emission spectrum of HARGO.

Through the electrochemical oxidation of an aqueous NaOH solution under low voltage and low current conditions over an extended period, we successfully synthesized a novel type of graphene oxide (GO). Specifically, electrolysis was conducted using a graphite rod (5 mm in diameter) as the anode and a platinum (Pt) electrode as the cathode in a 0.5 M NaOH aqueous solution for 96 hours at a voltage of 3.5 V and a current of 0.03 A. The resulting GO mixed aqueous solution is brownish, uniformly dispersed, and exhibits no noticeable precipitation (**Figure 1a**). Following dialysis and purification, GO powder was obtained through drying. The GO powder was dissolved in water, and following ultrasonic dispersion, GO aqueous solutions with concentrations of 10, 5, and 1 mg/mL were prepared (**Figure 1a**). To observe the microstructural characteristics of GO prepared by this method, atomic force microscopy (AFM) analysis was performed. The AFM results indicate that the lamellar thickness of GO prepared by this method is 0.414 nm. The morphology is elongated, with a length-to-width ratio of approximately 8:1, and it exhibits longitudinal cracks along the long axis (**Figure 1b**). Given the unique high aspect ratio morphology and structural characteristics of this GO, which differ from those of ordinary GO, it is designated as high aspect ratio GO (HARGO). The morphology of HARGO may result from the uniform oxidation of graphite under low voltage and current conditions, where the current flows from top to bottom along the grinding rod, leading to the characteristic strip shape of GO with a multi-rift structure.

The HARGO synthesized in this study demonstrates unique physicochemical properties compared to GO prepared by traditional methods, such as the modified Hummers method and rapid electrochemical oxidation method (Abdelkader et al., 2014; Korucu et al., 2023; Pei et al., 2018; Xu et al., 2023). UV-Vis analysis revealed a prominent absorption peak at 192 nm, along with three shoulder peaks at 225 nm, 260 nm, and 295 nm in the aqueous solution of HARGO. The absorption peaks at 192 nm, 225 nm, and 260 nm are specifically attributed to the characteristic π-π* transitions of HARGO, whereas the peak at 295 nm corresponds to n-π* transitions (**Figure 1c**). Fourier transform infrared spectrum (FT-IR) analysis identified absorption peaks for HARGO at 3440 cm^-1^, 2069 cm^-1^, 1637 cm^-1^, 1387 cm^-1^, 624 cm^-1^, and 557 cm^-1^. The absorption peaks at 1637 cm^1^ and 1387 cm^1^ indicate the presence of carboxyl groups (COO^-^), while the peak at 3440 cm^1^ signifies the existence of hydroxyl groups (-OH). The absence of a peak at 1720 cm^1^ suggests a lack of free carbonyl groups at the edges (**Figure 1d**). Zeta potential test results indicate that the content of oxygen-containing groups per unit area on the surface of HARGO (pH = 7, ζ = -8.5 mV) is lower than that of GO prepared by the traditional Hummers method (pH = 7, ζ = -27.5 mV) (**Figure 1e**). Titration test results indicate that the pKa of the carboxyl group in HARGO is approximately 4.82, the concentration of the carboxyl group is about 7.8 × 10^-3^ mol/g, and the surface charge per unit mass is approximately -751 C/g (**Figure 1f**), which is significantly higher than that of GO prepared by the traditional Hummers method (Korucu et al., 2023).

Interestingly, when ultraviolet light irradiates the HARGO aqueous solution, it is observed that HARGO emits fluorescence (**Figure 1g**). Subsequently, the excitation and emission spectra of the HARGO aqueous solution were analyzed. The excitation spectrum indicates that the maximum excitation wavelength of HARGO is 492 nm, with an excitation wavelength range of approximately 290–530 nm (**Figure 1h**). The emission spectrum reveals that the maximum emission wavelength of HARGO is 510 nm (**Figure 1i**).

### HARGO can significantly penetrate various tissues and cells in wheat seeds

**Figure 2:**
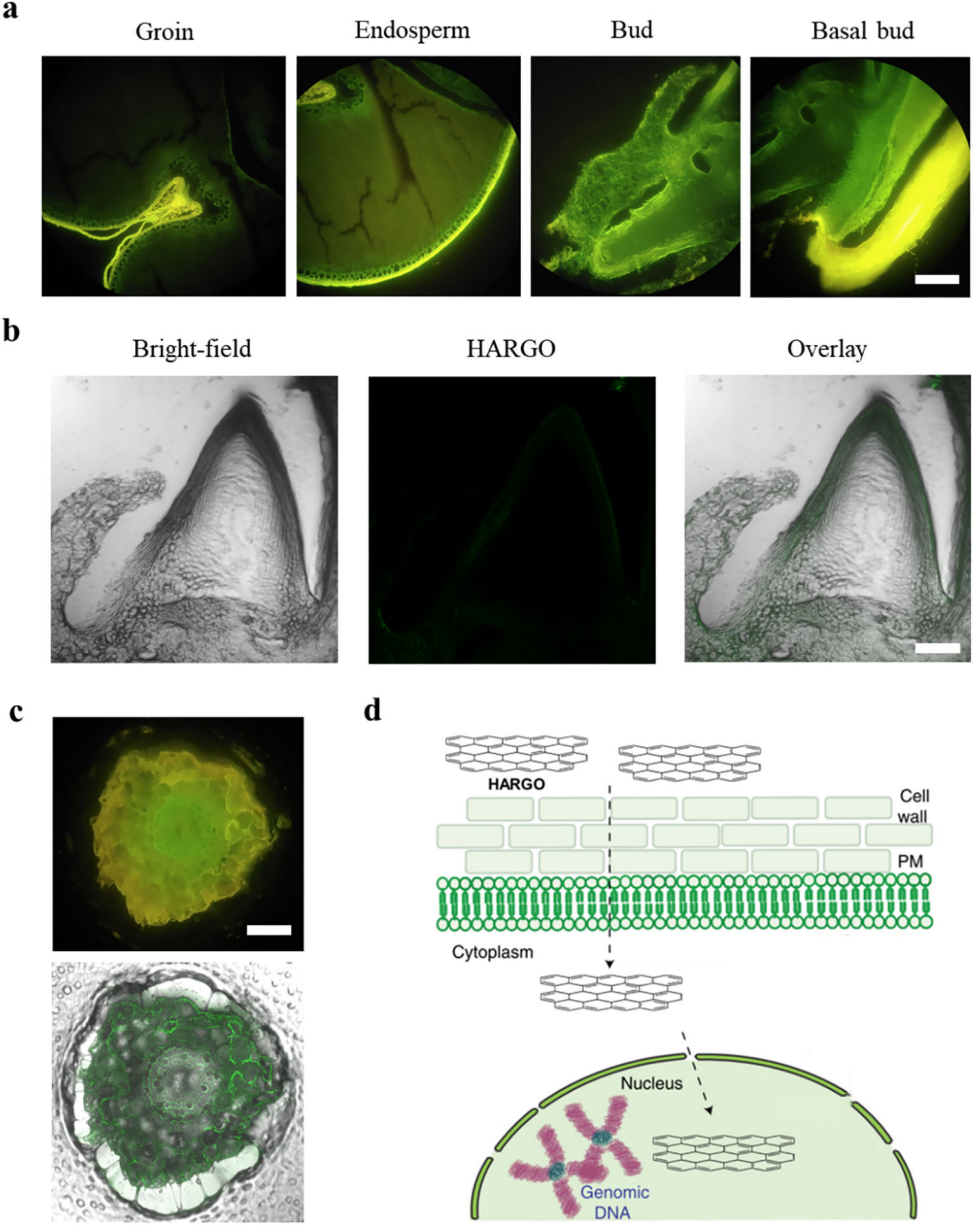
The distribution of high aspect ratio graphene oxide (HARGO) in various tissues and cells of wheat. **(a)**, Inverted fluorescence microscopy images of frozen sections of spring wheat Zhongkemai 138 (ZKM138) seeds, which were soaked in a 0.5 mg/mL HARGO aqueous solution for one day, depict the groin, endosperm, bud, and basal part of the embryo from left to right. The scale bar represents 25 µm. **(b)**, Laser confocal images of the bud are presented from left to right as bright field, HARGO, and overlay. The scale bar represents 25 µm. **(c)**, Inverted fluorescence microscopy image (top) and laser confocal microscopy image (bottom) of frozen cross-sections of wheat roots after 14 days post-seedling emergence. The scale bar represents 25 µm. **(d)**, Schematic representation of HARGO penetrating plant cells.

By utilizing the fluorescent properties of HARGO, we evaluated its ability to penetrate plant seeds. We used spring wheat Zhongkemai 138 (ZKM138) seeds and soaked them in a HARGO aqueous solution for 24 hours before performing frozen sectioning. To ensure that the plant cells were alive during observation, we examined all samples immediately after frozen sectioning. Inverted fluorescence microscopy results indicated that a substantial amount of HARGO was detected in the groin, endosperm, germ, and germ base of wheat seeds, while the seeds without HARGO immersion did not emit fluorescence (**Figure 2a**; **Supplementary Figure S1**). Further analysis using laser confocal microscopy revealed that HARGO was abundantly distributed in the germ cambium, establishing a basis for delivering plasmid DNA to plant seeds and subsequently generating positive seedlings (**Figure 2b**). To investigate whether HARGO penetrates the interior of plant cells, the roots of wheat seedlings soaked in HARGO aqueous solution were subjected to frozen sectioning and microstructural analysis. Inverted fluorescence microscopy revealed a substantial amount of HARGO in the roots, and laser confocal optical sectioning demonstrated that HARGO was not only significantly enriched on the cell wall but also present in large quantities within the cells (**Figure 2c**). Based on our experimental results, we drew a schematic diagram showing how HARGO penetrates the cell wall and enters plant cells (**Figure 2d**).

A preliminary assessment of the biological toxicity of HARGO was conducted using ZKM138 seeds. Seeds were co-cultured with an aqueous HARGO solution at a concentration of 0.5 mg/mL. The seeds in both the pure water group and the GO group were able to germinate normally (**Supplementary Figure S2a, c**), which indicated that HARGO exhibited no cytotoxic effects on ZKM138. Interestingly, HARGO significantly promoted the growth of seedlings and roots (**Supplementary Figures 2a–d**). The above results indicated that HARGO has high safety and good biocompatibility.

For a nanomaterial to achieve high penetrability into various plant tissues, it typically requires suitable size (including small particle size and uniform size distribution), favorable surface characteristics (including appropriate surface charge, good hydrophilicity or hydrophobicity, as well as surface modification and functionalization), adequate dispersibility, good biocompatibility, and high stability (Kranjc and Drobne, 2019). HARGO exhibits a distinctive morphological structure, favorable surface characteristics, high dispersibility, and remarkable stability (**Figures 1**, **2**; **Supplementary Figure 2**). According to above, the HARGO has significant advantages for applications in bio-material science.

### Adsorption capacity of HARGO for plasmid DNA pEG100-PcNAC2-EGFP

It is widely reported that GO can effectively adsorb plasmid DNA. The specific adsorption mechanism encompasses several physicochemical properties, including local electrostatic attraction, hydrogen bonding between carboxyl, hydroxyl, and epoxy groups with the amino group of plasmid DNA, van der Waals interactions, flexible wrapping of plasmid DNA by GO, and π-π stacking between the bases in GO and plasmid DNA (Kim et al., 2015; Lei et al., 2011).

**Figure 3:**
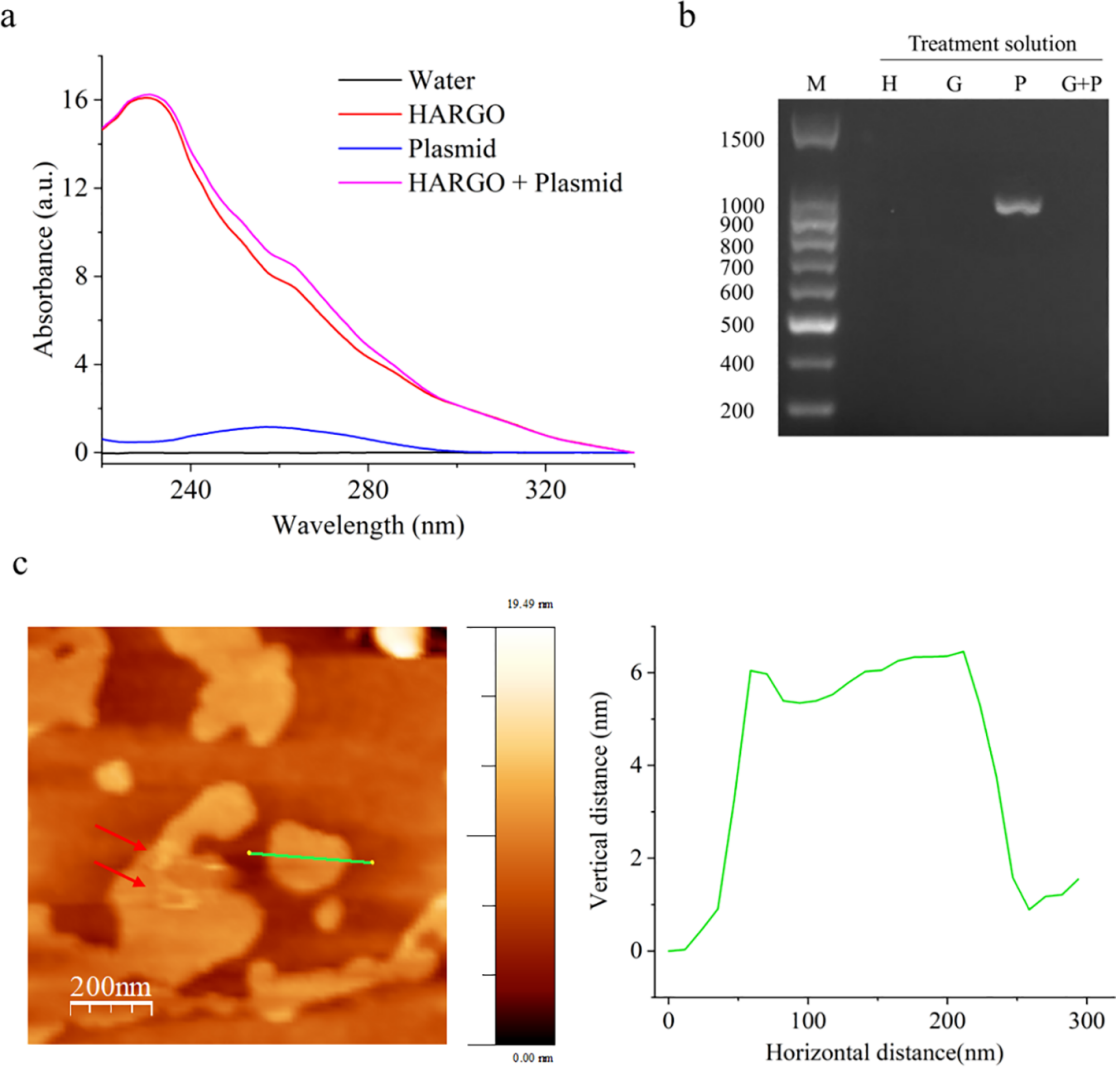
High aspect ratio graphene oxide (HARGO) adsorbs plasmid DNA pEG100-PcNAC2-EGFP. **(a)**, UV spectra of water, HARGO, plasmid DNA pEG100-PcNAC2-EGFP, and mixed aqueous solutions of HARGO and pEG100-PcNAC2-EGFP. **(b)**, Digital photographs of agarose gel electrophoresis after PCR amplification were obtained using water (H), HARGO (G), plasmid DNA pEG100-PcNAC2-EGFP (P), and their mixed aqueous solution (G+P) as templates. **(c)**, The left image displays an atomic force microscopy image of a mixed aqueous solution of HARGO and pEG100-PcNAC2-EGFP, with arrows indicating the presence of pEG100-PcNAC2-EGFP. The right image depicts the height profile. The stacked HARGO may be attributed to the fact that the pH of the solution in the state is close to 7, leading to a decrease in the repulsive force between graphene oxide layers.

Given the excellent penetrability of HARGO into plant tissues, its effective binding to plasmid DNA is essential for successful cargo delivery. To investigate HARGO’s adsorption capacity for plasmid DNA, we first constructed the plasmid DNA pEG100-PcNAC2-EGFP, which is 11, 798 bp in length (**Supplementary Figure 3**). Subsequently, we established four experimental groups: a water-only control group, a HARGO-only group, a pEG100-PcNAC2-EGFP-only group, and a mixed group containing both HARGO and pEG100-PcNAC2-EGFP. UV-Vis analysis revealed that in the mixed group of HARGO and pEG100-PcNAC2-EGFP, the absorbance value at 225 nm was 16.24, significantly lower than the combined absorbance values of the HARGO-only group (16.11) and the pEG100-PcNAC2-EGFP-only group (0.49), resulting in an expected value of 16.60. Furthermore, compared to the HARGO-only group, the mixed group exhibited a redshift in the absorbance peak at 225 nm, suggesting that HARGO may have physically adsorbed plasmid DNA (**Figure 3a**).

To confirm the physical adsorption of HARGO to pEG100-PcNAC2-EGFP, a PCR analysis was conducted. The results indicated that the mixture of HARGO and pEG100-PcNAC2-EGFP did not amplify the target DNA band, suggesting that physical adsorption occurred between HARGO and the plasmid DNA. This adsorption impeded the attachment of DNA polymerase to the plasmid DNA adsorbed on the HARGO surface (**Figure 3b**). Lastly, we employed AFM to assess the status of HARGO adsorbing pEG100-PcNAC2-EGFP. The results clearly confirmed that HARGO, similar to conventional GO, can effectively adsorb plasmid DNA through physical adsorption. (**Figure 3c**).

### HARGO-mediated delivery of pEG100-PcNAC2-EGFP plasmid DNA to *Poa crymophila* Keng

The above results of the research indicate that HARGO exhibits high penetrability, allowing it to penetrate various tissues and cells of wheat while also physically adsorbing plasmid DNA. Based on these findings, we hypothesize that HARGO may possess significant potential for delivering plasmid DNA into plant seeds.

**Figure 4:**
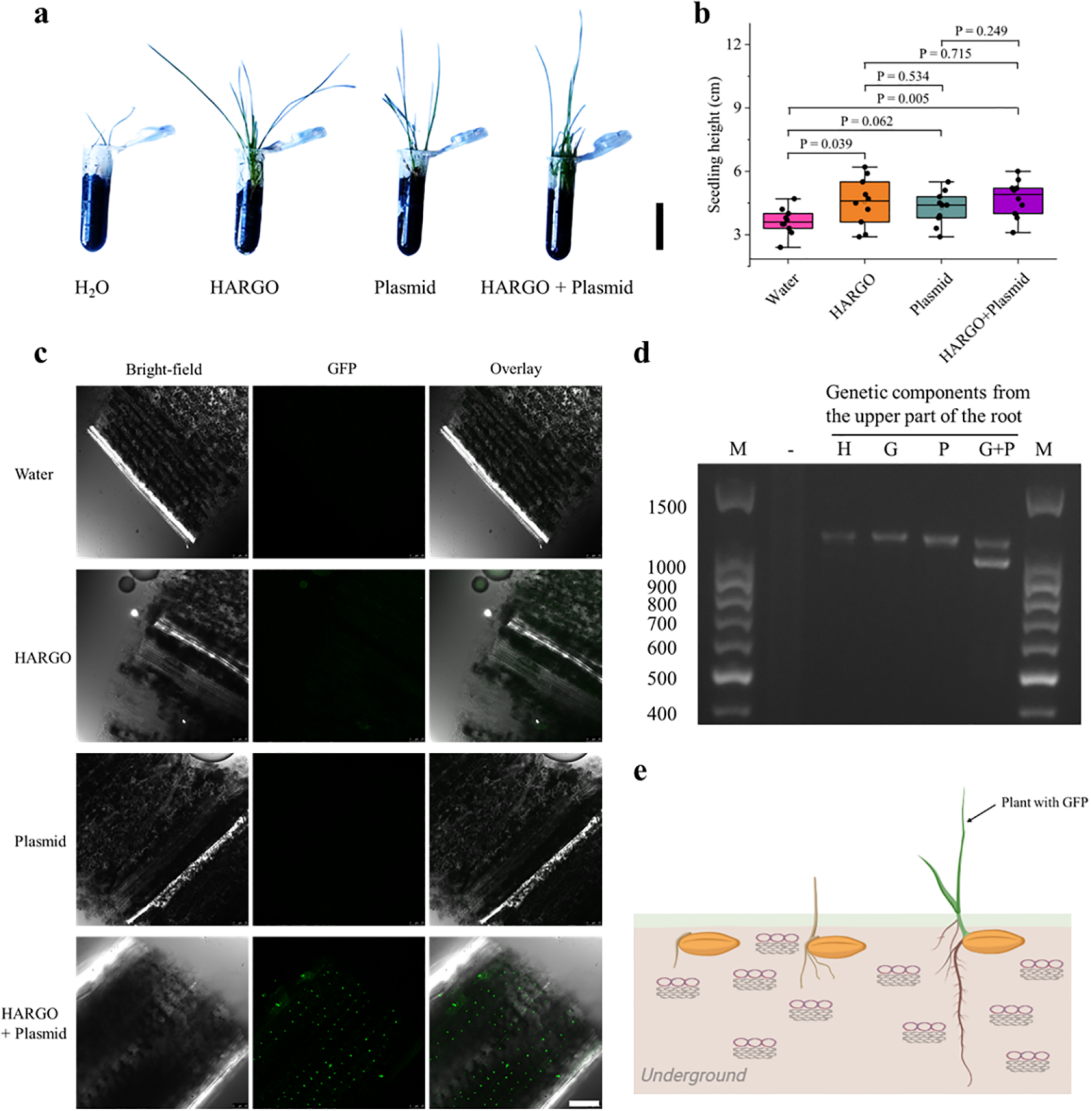
High aspect ratio graphene oxide (HARGO) was used to deliver plasmid DNA pEG100-PcNAC-EGFP to the seeds of *Poa crymophila* Keng and produce positive plants. **(a)**, A comparison of irrigation using water, HARGO, plasmid DNA pEG100-PcNAC2-EGFP, and a mixed aqueous solution of HARGO and pEG100-PcNAC2-EGFP, with digital photographs taken 14 days after the emergence of *Poa crymophila* Keng seedlings in dry soil. The scale bar represents 2.5 cm. **(b)**, Statistical analysis of the seedling lengths of ten randomly selected *Poa crymophila* Keng seedlings. **(c)**, Laser confocal microscopy images of the leaf tips from four groups of *Poa crymophila* Keng. The scale bar represents 25 µm. **(d)**, Agarose gel electrophoresis images of PCR products from four groups of *Poa crymophila* Keng seedlings: water (H), HARGO (G), plasmid DNA pEG100-PcNAC2-EGFP (P), and a mixed aqueous solution of HARGO and pEG100-PcNAC2-EGFP (G+P). e, Schematic representation of HARGO delivering plasmid DNA to plant seeds and resulting in positive seedlings.

We further investigated whether HARGO can effectively deliver plasmid DNA to plant seeds, using *Poa crymophila* Keng cv. Qinghai seeds. Previously, the plasmid pEG100-PcNAC2-EGFP has been experimentally validated to enable stable expression of the fusion protein PcNAC2-EGFP in *Poa crymophila* Keng cv. Qinghai seeds (Li et al., 2025). The seeds were sown in 2-milliliter Eppendorf tubes containing a dry matrix soil at a density of 20 seeds per tube and irrigated with water, HARGO aqueous solution, plasmid DNA pEG100-PcNAC2-EGFP aqueous solution, and a HARGO + pEG100-PcNAC2-EGFP mixed aqueous solution, respectively. After 14 days of seedling emergence, we observed the seedlings (**Figure 4a**), and the statistical analysis of seedling height indicated that both the HARGO aqueous solution and the HARGO + pEG100-PcNAC2-EGFP mixed aqueous solution significantly promoted seedling growth (**Figure 4b**).

We performed frozen sectioning on the apical portions of the seedlings, followed by immediate laser confocal detection. The results indicated that no positive signals of green fluorescent protein (GFP) were detected in the Water, HARGO, and Plasmid groups (**Figure 4c**). In contrast, the HARGO + Plasmid group exhibited positive GFP signals (**Figure 4c**; **Supplementary Figure 5**). To further verify the delivery of plasmid DNA pEG100-PcNAC2-EGFP to *Poa crymophila* Keng cv. Qinghai by HARGO, we conducted PCR analysis on seedlings from the four treatment groups. Consequently, we successfully amplified a specific band corresponding to pEG100-PcNAC2-EGFP in the seedlings from the HARGO + Plasmid group, measuring 978 bp (**Figure 4d**), which indicates the presence of plasmid DNA pEG100-PcNAC2-EGFP in the seedlings of *Poa crymophila* Keng cv. Qinghai. Finally, to assess the success rate of HARGO in delivering plasmid DNA, we randomly performed cryosectioning and laser confocal detection on the apical parts of 10 *Poa crymophila Keng* cv. Qinghai seedlings (**Supplementary Figure 4**). We found that 9 seedlings exhibited positive GFP signals, indicating a 90% success rate for HARGO in plasmid DNA delivery (**Supplementary Figure 4**).

Traditional graphene oxide can adsorb plasmid DNA, but it cannot directly transfer the DNA into plant cells or various plant tissues. To enable GO to deliver plasmid DNA to plant cells, it must be modified with PEG—primarily to enhance biocompatibility—and PEI—mainly to improve the physical adsorption between GO and plasmid DNA (Feng et al., 2013). However, since both PEG and PEI are macromolecules, the physical volume of the GO-PEG-PEI complex is substantial, hindering its ability to deliver plasmid DNA to the deeper layers of plant tissues—only allowing delivery to the surface layers (Feng et al., 2013). In this study, HARGO can effectively adsorb plasmid DNA and transport it to the deep layers of plant tissues without the need for functionalization treatment with PEG or PEI (**Figures 3**, **4e**). This is likely attributed to the small volume of the HARGO-plasmid DNA complex. Additionally, it may benefit from the elongated morphology of the HARGO-plasmid DNA complex, analogous to that of carbon nanotubes and silicon carbide whiskers (Demirer et al., 2019; Matsushita et al., 1999).

## Discussion

### Challenges and optimization strategies of HARGO for seed-mediated DNA delivery

Although HARGO exhibits high efficiency for seed-mediated DNA delivery, several limitations still remain. First, the preparation of graphene oxide nanosheets is time-consuming. Ultrathin sheets (~0.4 nm) can only be produced via oxidation under strictly low voltage and low current conditions. Second, seed soaking requires a large dosage of HARGO, resulting in high material consumption and increased experimental costs. Third, this system demands a considerable amount of plasmid DNA; seed treatment generally requires several milliliters of plasmid solution at a concentration of 100 ng/μL, which further raises the overall experimental cost. Moreover, the delivery efficiency cannot reach 100%, and it remains unclear why a small proportion of individual plants fail to take up plasmid DNA.

To overcome these bottlenecks, corresponding optimization strategies can be implemented. Simplified and high-efficiency synthesis routes should be explored to shorten the preparation cycle of ultrathin GO nanosheets and eliminate the dependence on harsh low-voltage and low-current oxidation conditions. Surface functional modification of HARGO is also feasible to strengthen its binding capacity with nucleic acids and plant tissues, enabling effective DNA delivery at reduced working concentrations and thus lowering GO consumption. In addition, the soaking system can be further optimized by introducing absorption-promoting adjuvants and miniaturizing the treatment volume, which greatly decreases the required dosage and volume of high-concentration plasmid solution. Furthermore, clarifying the interaction mechanism between HARGO–plasmid complexes and plant cells, as well as optimizing key treatment parameters such as soaking duration and incubation temperature, will help eliminate individual differences among plant materials and ultimately improve the consistency and overall efficiency of seed-mediated DNA delivery.

### Selection of *PcNAC2* gene plasmid as a model for HARGO-mediated intracellular delivery

In this study, the plasmid carrying the *PcNAC2* gene was selected as the model vector for intracellular delivery. This choice was guided by practical and experimental considerations aligned with our primary objective: to validate that the HARGO-based system can effectively replace conventional gene delivery tools. First, the plasmid pEG100-PcNAC2-EGFP has been previously demonstrated to successfully transform plant cells and exhibits stable transfection capability in plant systems (Li et al., 2025). Second, the gene carried by this plasmid is efficiently transcribed in plant cells and translated into functional PcNAC protein, thereby eliminating potential interference from untranscribed genes or unstable plasmids. Third, the transfection of pEG100-PcNAC2-EGFP into *Poa crymophila* Keng utilizes a system readily accessible in our laboratory, allowing us to focus on evaluating the delivery performance of the graphene carrier without dedicating additional time to constructing and validating new gene-plasmid systems.

NAC (NAM, ATAF1/2, CUC2) is a conserved plant-specific transcription factor family that participates extensively in plant growth, development, stress response and metabolic regulation (Nuruzzaman et al., 2010; Xiong et al., 2025). Using the pEG100-PcNAC2-EGFP plasmid as a delivery model not only evaluated the delivery efficiency of HARGO, but also laid a foundation for subsequent functional analysis of PcNAC2, as efficient intracellular delivery is essential for exploring gene biological functions. The NAC family proteins contain an N-terminal conserved nuclear localization signal (NLS), which mediates protein translocation from the cytoplasm to the nucleus (Ernst et al., 2004; Olsen et al., 2005). Nuclear entry is a prerequisite for NAC protein to exert transcriptional regulation, allowing it to bind target gene promoters and modulate gene expression. Laser confocal microscopy confirmed that the PcNAC2 plasmid could be normally expressed in plant cells after HARGO-mediated delivery, further verifying the reliability and efficacy of the reduced graphene oxide-based intracellular delivery system.

### Versatile applications of GO in multiple fields

Graphene Oxide (GO), featuring its unique two-dimensional structure, high specific surface area, abundant oxygen-containing functional groups, and modifiability, has become a research hotspot across multiple disciplines. Its applications cover antibacterial fields, biomedicine, environmental governance, and energy storage, with distinct advantages and broad prospects in each field.

In the field of biomedicine, GO shows remarkable application value, especially in antibacterial and drug delivery aspects. The antibacterial effect of GO mainly relies on the synergistic mechanism of physical membrane damage and chemical oxidative stress: its sharp edges can directly cut bacterial membranes, while the oxygen-containing groups on its surface induce the accumulation of reactive oxygen species (ROS), leading to lipid extraction and loss of membrane integrity (Akhavan and Ghaderi, 2010; Liu et al., 2011). Studies have shown that GO nanosuspensions can inhibit common pathogenic bacteria such as Escherichia coli with an inhibition rate exceeding 90%, and exhibit low toxicity to mammalian cells (Singh et al., 2025; Tu et al., 2013). In terms of drug delivery, leveraging π–π stacking and electrostatic interactions, GO can efficiently load chemotherapeutic drugs such as doxorubicin, with a drug loading capacity of up to 200% (weight ratio), and achieve targeted release of tumors through targets such as folate receptors, which significantly improves the therapeutic effect of chemotherapy and reduces side effects (Yang et al., 2008; Zhao and Liu, 2014).

In the environmental field, GO mainly exerts efficient adsorption, membrane separation, and photocatalytic functions. Its large specific surface area and hydrophilicity endow it with strong adsorption capacity for heavy metal ions and organic dyes (Narayanaswamy et al., 2020; Zhao et al., 2011); GO membranes can achieve a desalination efficiency of over 90% in seawater desalination (Li et al., 2018; Tiwary et al., 2024). In addition, the composite of GO with semiconductors such as TiO_2_ can degrade organic pollutants under photocatalysis, providing an efficient and environmentally friendly solution for environmental remediation (Khalid et al., 2013; Tang et al., 2018).

GO and its derivatives also perform prominently in energy storage. As electrode materials for supercapacitors, graphene derivative-based devices can sustain over 1000 charge–discharge cycles, demonstrating outstanding long-term cycling stability (Tene et al., 2024). In lithium-ion batteries, graphene-based composites (such as MnO_2_/graphene and silicon/graphene) can significantly enhance the specific capacity and rate performance of electrodes, while effectively suppressing volume expansion and resistance growth during cycling (Abdi et al., 2025; Liu et al., 2022). These characteristics make them highly promising for application in the development of high-performance energy storage devices.

### Recent progress in GO and its derivatives for gene delivery

In recent years (2025–2026), substantial advances have been achieved in the application of chemically modified and functionally decorated graphene oxide (GO) and its derivatives in gene delivery and related fields, covering tumor therapy, orthopedic repair, mechanism research and delivery system optimization. In tumor gene therapy, functionally modified small-sized GO (smGO) can co-deliver siRNA and pDNA in 3D lung cancer models, achieving efficient multi-gene synergistic clearance (Grilli et al., 2025); GO-based nano-systems can also deliver anti-miR-21 antisense oligonucleotides in *in vivo* liver cancer models, providing experimental evidence for targeted nucleic acid delivery (Trischitta et al., 2026). In orthopedic repair, GO-PEG/pBMP-2 complexes coated on sulfonated PEEK implants realize localized and sustained osteogenic gene delivery, achieving integrated “osteogenesis-antibacterial” dual functions (Pan et al., 2026). The research published in *Nanoscale* systematically explored how the topological structure and grafting density of polyethyleneimine (PEI) affect the binding behavior of GO to DNA, revealing the molecular mechanism of the carrier-nucleic acid interaction in non-viral gene delivery, and providing a theoretical basis for the rational design of efficient and low-toxic GO-PEI gene vectors (Davoudinia et al., 2025). An immobilized micropatterned reduced graphene oxide (rGO) chip, integrated with a 1064 nm nanosecond pulsed laser, enables the high-throughput optical perforation of approximately 5×10^5^ cells within 15 seconds, facilitating the efficient and uniform intracellular delivery of plasmids, siRNAs, and macromolecular enzymes (Dominic et al., 2026).

## Conclusion

This study successfully developed a novel type of graphene oxide, termed HARGO, using a low-voltage, low-current, long-duration electrochemical oxidation method. HARGO demonstrates significant penetration into plant tissues, efficiently adsorbing and delivering plasmid DNA to *Poa crymophila* Keng seeds, leading to the production of transgenic plants with a transformation efficiency of up to 90%.

Compared to traditional plasmid DNA delivery methods—such as *Agrobacterium*-mediated transfer, gene guns, electroporation, and plant viral delivery methods—HARGO serves as a delivery carrier that offers distinct advantages, including high speed, simplicity, safety, high transformation efficiency, and low cost. Given its unique properties, HARGO is anticipated to play a crucial role in areas such as plant transgenesis, gene editing, and gene knockout, thereby facilitating crop improvement and enhancing agricultural production. Furthermore, considering HARGO’s capability to penetrate tissue cells effectively, it holds significant potential in the medical field and may be employed for tumor-targeted therapy, treatment of genetic diseases, and gene therapy.

Ultimately, the high aspect ratio and penetration capability of HARGO present new opportunities for its development as a nanomedicine carrier, particularly in improving drug delivery efficiency and targeting precision. In conclusion, this study has successfully synthesized GO with a high aspect ratio and demonstrated its potential applications in tissue culture-free plant genetic transformation. As a novel class of nanocarriers, HARGO holds significant promise for future applications in biotechnology and medicine, warranting further research and development.

## Data Availability

The original contributions presented in the study are included in the article/**Supplementary Material**. Further inquiries can be directed to the corresponding author.

## Supplementary material

Supplementary Figure 1 Inverted fluorescence microscopy images of the leaf sections of spring wheat Zhongkemai 138 (ZKM138). Plants do not inherently emit fluorescence. The scale bar represents 50 µm.

Supplementary Figure 2 Effect of high aspect ratio graphene oxide (HARGO) on wheat seedlings. **(a, b)**, Randomly select 15 seeds of spring wheat variety Zhongkemai 138 (ZKM138) and place them in a culture dish for germination. Digital photo of wheat seedlings 14 days after emergence **(a)** and their height statistics **(b)**. Among them, the number of seedlings in both groups is 45. **(c, d),** Digital photo of roots remaining in the culture medium after harvesting wheat seedlings **(c)** and statistics of root fresh weight **(d)**.

Supplementary Figure 3 The gene information of pEG100-PcNAC2-EGFP. **(a)**, Plasmid map of pEG100-PcNAC2-EGFP. **(b),** The sequence of PcNAC2-EGFP. The sequence with a yellow background is the cDNA of PcNAC2 gene, the blue background represents a restriction site (XhoI), the green background shows EGFP, and the gray background indicates 6×HA.

Supplementary Figure 4 Laser confocal microscopy images of ten 21-day-old *Poa crymophila* Keng seedlings. **(a–j),** Confocal fluorescence microscopy images of seedlings germinated from ten independent *Poa crymophila* Keng seeds following the treatment with HARGO and plasmid solution. As shown in **(b)**, one seedling exhibited no detectable fluorescence; thus, the transfection efficiency was 90%. The excitation wavelength is 488 nm. The scale bar length is 50 µm.

Supplementary Figure 5 Laser confocal microscopy images of the GFP-positive leaf tips. The yellow-framed image shows a magnified view of the region circled in the Overlay panel. A single cell is outlined by the yellow dashed box, and the green signal marked by the white arrow is likely derived from chloroplast autofluorescence. The scale bar represents 25 µm.

## References

[B1] Abdelkader A. M. Kinloch I. A. Dryfe R. A. W. (2014). High-yield electro-oxidative preparation of graphene oxide. Chem. Commun. 50, 8402–8404. doi: 10.1039/c4cc03260h 24948081

[B2] Abdi A. Sarraf-Mamoory R. Stich M. Baumer C. Ullmann F. Krischok S. . (2025). Optimization of in situ hydrothermal synthesis of birnessite MnO2/graphene composite: thermodynamic insights and enhanced electrochemical performance for Li-ion battery anodes. J. Mater. Sci. - Mater. Electron. 36, 1750. doi: 10.1007/s10854-025-15800-0 30311153

[B3] Abrahamian P. Hammond R. W. Hammond J. (2020). Plant virus-derived vectors: Applications in agricultural and medical biotechnology. Annu. Rev. Virol. 7, 513–535. doi: 10.1146/annurev-virology-010720-054958 32520661

[B4] Akhavan O. Ghaderi E. (2010). Toxicity of graphene and graphene oxide nanowalls against bacteria. ACS Nano 4, 5731–5736. doi: 10.1021/nn101390x 20925398

[B5] Azizi-Dargahlou S. Pouresmaeil M. (2024). Agrobacterium tumefaciens-mediated plant transformation: A review. Mol. Biotechnol. 66, 1563–1580. doi: 10.1007/s12033-023-00788-x 37340198

[B6] Cai Y. Liu Z. Wang H. Meng H. Cao Y. (2024). Mesoporous silica nanoparticles mediate siRNA delivery for long-term multi-gene silencing in intact plants. Advanced Sci. (Weinheim Baden-Wurttemberg Germany) 11, e2301358. doi: 10.1002/advs.202301358 38145358 PMC10916655

[B7] Chang F. P. Kuang L. Y. Huang C. A. Jane W. N. Hung Y. Hsing Y. C. . (2013). A simple plant gene delivery system using mesoporous silica nanoparticles as carriers. J. Mater. Chem. B 1, 5279–5287. doi: 10.1039/c3tb20529k 32263331

[B8] Chung S. M. Vaidya M. Tzfira T. (2006). Agrobacterium is not alone: gene transfer to plants by viruses and other bacteria. Trends Plant Sci. 11, 1–4. doi: 10.1016/j.tplants.2005.11.001 16297655

[B9] Davoudinia S. Imani R. Tafazzoli-Shadpour M. Fani N. Makki H. (2025). Tunable DNA binding on graphene oxide via polyethyleneimine topology and density: mechanistic insights for nonviral gene delivery. Nanoscale 17, 27911–27922. doi: 10.1039/d5nr03441h 41327575

[B10] Demirer G. S. Zhang H. Matos J. L. Goh N. S. Cunningham F. J. Sung Y. . (2019). High aspect ratio nanomaterials enable delivery of functional genetic material without DNA integration in mature plants. Nat. Nanotechnol. 14, 456–464. doi: 10.1038/s41565-019-0382-5 30804481 PMC10461892

[B11] Desai P. N. Shrivastava N. Padh H. (2010). Production of heterologous proteins in plants: strategies for optimal expression. Biotechnol. Adv. 28, 427–435. doi: 10.1016/j.bioteChadv.2010.01.005 20152894

[B12] Dominic D. Ojha R. Nagai M. Kar S. Santra T. S. (2026). Immobilized, micro-patterned graphene nanoflake devices for high-throughput, uniform intracellular biomolecular delivery. Mater. Adv. 7, 2279–2299. doi: 10.1039/d5ma01317h

[B13] Dong S. Jing X. Lin S. Lu K. Li W. Lu J. . (2022). Root hair apex is the key site for symplastic delivery of graphene into plants. Environ. Sci. Technol. 56, 12179–12189. doi: 10.1021/acs.est.2c01926 35947795

[B14] Dunbar T. Tsakirpaloglou N. Septiningsih E. M. Thomson M. J. (2022). Carbon nanotube-mediated plasmid DNA delivery in rice leaves and seeds 23. 10.3390/ijms23084081 PMC9028948 35456898

[B15] Eidenberger L. Kogelmann B. Steinkellner H. (2023). Plant-based biopharmaceutical engineering. Nat. Rev. Bioeng. 1, 426–439. doi: 10.1038/s44222-023-00044-6 37317690 PMC10030082

[B16] Ernst H. A. Olsen A. N. Larsen S. Lo Leggio L. (2004). Structure of the conserved domain of ANAC, a member of the NAC family of transcription factors. EMBO Rep. 5, 297–303. doi: 10.2210/pdb1ut4/pdb 15083810 PMC1299004

[B17] Etit D. Meramo S. Ögmundarson Ó. Jensen M. K. Sukumara S. (2024). Can biotechnology lead the way toward a sustainable pharmaceutical industry? Curr. Opin. Biotechnol. 87, 103100. doi: 10.1016/j.copbio.2024.103100 38471403

[B18] Feng L. Yang X. Shi X. Tan X. Peng R. Wang J. . (2013). Polyethylene glycol and polyethylenimine dual-functionalized nano-graphene oxide for photothermally enhanced gene delivery. Small (Weinheim An. der Bergstrasse Germany) 9, 1989–1997. doi: 10.1002/smll.201202538 23292791

[B19] Grilli F. Sakib S. Variola F. Zou S. (2025). Graphene oxide-based gene modulation in preferential elimination of lung cancer cells in a 3D tumor microenvironment model 5.

[B20] Khalid N. R. Ahmed E. Hong Z. Sana L. Ahmed M. (2013). Enhanced photocatalytic activity of graphene–TiO2 composite under visible light irradiation. Curr. Appl. Phys. 13, 659–663. doi: 10.1016/j.cap.2012.11.003 38826717

[B21] Kim S. Park C. Gang J. (2015). Effect of pH and salt on adsorption of double-stranded DNA on graphene oxide. J. Nanosci. Nanotechnol. 15, 7913–7917. doi: 10.1166/jnn.2015.11217 26726439

[B22] Korucu H. Mohamed A. I. Yartaşı A. Uğur M. (2023). The detailed characterization of graphene oxide. Chem. Pap. 77, 5787–5806. doi: 10.1007/s11696-023-02897-y 30311153

[B23] Kranjc E. Drobne D. (2019). Nanomaterials in plants: a review of hazard and applications in the agri-food sector. Nanomaterials (Basel Switzerland) 9, 1094. doi: 10.3390/nano9081094 31366106 PMC6723683

[B24] Lei H. Mi L. Zhou X. Chen J. Hu J. Guo S. . (2011). Adsorption of double-stranded DNA to graphene oxide preventing enzymatic digestion. Nanoscale 3, 3888–3892. doi: 10.1039/c1nr10617a 21829836

[B25] Li S. Li J. Du M. Deng G. Song Z. Han H. (2022). Efficient gene silencing in intact plant cells using siRNA delivered by functional graphene oxide nanoparticles. Angewandte Chemie (International Ed. English) 61, e202210014. doi: 10.1002/ange.202210014 35921481

[B26] Li W. Wu W. Li Z. (2018). Controlling interlayer spacing of graphene oxide membranes by external pressure regulation. ACS Nano 12, 9309–9317. doi: 10.1021/acsnano.8b04187 30183255

[B27] Li X. Y. Ma Y. B. Li W. L. Li J. H. Li M. J. Li C. X. . (2025). Overexpression of PcNAC2 from Poa crymophila significantly enhances plant tolerance to chilling, salinity and osmotic stresses. BMC Plant Biol. 25, 1600. doi: 10.1186/s12870-025-07611-6 41257579 PMC12628889

[B28] Li Y. Yuan H. von dem Bussche A. Creighton M. Hurt R. H. Kane A. B. . (2013). Graphene microsheets enter cells through spontaneous membrane penetration at edge asperities and corner sites. PNAS 110, 12295–12300. doi: 10.1073/pnas.1222276110 23840061 PMC3725082

[B29] Liu Z. Tian Y. Wang P. Zhang G. (2022). Applications of graphene-based composites in the anode of lithium-ion batteries 4, 952200. doi: 10.3389/fnano.2022.952200

[B30] Liu Q. Yang F. Zhang J. Liu H. Rahman S. Islam S. . (2021). Application of CRISPR/Cas9 in crop quality improvement. Int. J. Mol. Sci. 22, 4206. doi: 10.3390/ijms22084206 33921600 PMC8073294

[B31] Liu S. Zeng T. H. Hofmann M. Burcombe E. Wei J. Jiang R. . (2011). Antibacterial activity of graphite, graphite oxide, graphene oxide, and reduced graphene oxide: membrane and oxidative stress. ACS Nano 5, 6971–6980. doi: 10.1021/nn202451x 21851105

[B32] Lv Z. Jiang R. Chen J. Chen W. (2020). Nanoparticle-mediated gene transformation strategies for plant genetic engineering. Plant Journal: For. Cell. Mol. Biol. 104, 880–891. doi: 10.1111/tpj.14973 32860436

[B33] Matsushita J. Otani M. Wakita Y. Tanaka O. Shimada T. (1999). Transgenic plant regeneration through silicon carbide whisker-mediated transformation of rice (Oryza sativa L.). Breed. Sci. 49, 21–26. doi: 10.1270/jsbbs.49.21

[B34] Mitter N. Worrall E. A. Robinson K. E. Li P. Jain R. G. Taochy C. . (2017). Clay nanosheets for topical delivery of RNAi for sustained protection against plant viruses. Nat. Plants 3, 16207. doi: 10.1038/nplants.2016.207 28067898

[B35] Narayanaswamy V. Alaabed S. Al-Akhras M. A. Obaidat I. M. (2020). Molecular simulation of adsorption of methylene blue and rhodamine B on graphene and graphene oxide for water purification. Mater. Today Proc. 28, 1078–1083. doi: 10.1016/j.matpr.2020.01.086 38826717

[B36] Nuruzzaman M. Manimekalai R. Sharoni A. M. Satoh K. Kondoh H. Ooka H. . (2010). Genome-wide analysis of NAC transcription factor family in rice. Gene 465, 30–44. doi: 10.1016/j.gene.2010.06.008 20600702

[B37] Olsen A. N. Ernst H. A. Leggio L. L. Skriver K. (2005). NAC transcription factors: structurally distinct, functionally diverse. Trends Plant Sci. 10, 79–87. doi: 10.1016/j.tplants.2004.12.010 15708345

[B38] Pan Y. Yiminjiang B. Abula A. Tuerxun R. Aishan M. (2026). Dual-functional sulfonated PEEK implants via graphene oxide–mediated BMP-2 gene delivery: enhanced osteogenic and antibacterial performance *in vitro* 13, 1763692. doi: 10.3389/fmed.2026.1763692 PMC12996093 41859135

[B39] Pawar P. Anumalla S. Sharma S. (2023). Role of carbon nanotubes (CNTs) in transgenic plant development. Biotechnol. Bioeng. 120, 3493–3500. doi: 10.1002/bit.28550 37691181

[B40] Pei S. Wei Q. Huang K. Cheng H. M. Ren W. (2018). Green synthesis of graphene oxide by seconds timescale water electrolytic oxidation. Nat. Commun. 9, 145. doi: 10.1038/s41467-025-65021-6 29321501 PMC5762692

[B41] Peng L. Xu Z. Liu Z. Wei Y. Sun H. Li Z. . (2015). An iron-based green approach to 1-h production of single-layer graphene oxide. Nat. Commun. 6, 5716. doi: 10.1038/ncomms6716 25607686 PMC4354147

[B42] Pradhan A. Mbohwa C. (2014). Development of biofuels in South Africa: challenges and opportunities. Renewable Sustain. Energy Rev. 39, 1089–1110. doi: 10.1016/j.rser.2014.07.131 38826717

[B43] Reid W. V. Ali M. K. Field C. B. (2020). The future of bioenergy. Global Change Biol. 26, 274–286. doi: 10.1111/gcb.14883 31642554 PMC6973137

[B44] Singh M. P. Kumar A. Singh A. Singh S. S. Chaurasia S. K. (2025). Surface-engineered graphene oxide–MXene–SLG composite with enhanced bactericidal properties 26, 20.

[B45] Squire H. J. Tomatz S. Voke E. González-Grandío E. Landry M. (2023). The emerging role of nanotechnology in plant genetic engineering. Nat. Rev. Bioeng. 1, 314–328. doi: 10.1038/s44222-023-00037-5 37880705

[B46] Su W. Xu M. Radani Y. Yang L. (2023). Technological development and application of plant genetic transformation 24. 10.3390/ijms241310646 PMC10341552 37445824

[B47] Tang B. Chen H. Peng H. Wang Z. Huang W. (2018). Graphene modified TiO_2_ composite photocatalysts: mechanism, progress and perspective. Nanomaterials (Basel Switzerland) 8, 105. doi: 10.3390/nano8020105 29439545 PMC5853736

[B48] Tene T. Bellucci S. Guevara M. Romero P. Guapi A. Gahramanli L. . (2024). Role of graphene oxide and reduced graphene oxide in electric double-layer capacitors: A systematic review 10.

[B49] Tiwary S. K. Singh M. Chavan S. V. Karim A. (2024). Graphene oxide-based membranes for water desalination and purification. NPJ 2D Mater. Appl. 8, 27. doi: 10.1038/s41699-024-00462-z 37880705

[B50] Trischitta P. Nasiłowska B. Pennisi R. Costa M. Sciortino M. T. Kutwin M. (2026). Graphene oxide–antisense miR-21 nanosystem modulates gene expression and suppresses tumorigenesis in HepG2-derived CAM xenografts 16, 523. doi: 10.3390/biom16040523 PMC13113956 42072644

[B51] Tu Y. Lv M. Xiu P. Huynh T. Zhang M. Castelli M. . (2013). Destructive extraction of phospholipids from Escherichia coli membranes by graphene nanosheets. Nat. Nanotechnol. 8, 594–601. doi: 10.1038/nnano.2013.125 23832191

[B52] Tyczewska A. Twardowski T. Woźniak-Gientka E. (2023). Agricultural biotechnology for sustainable food security. Trends Biotechnol. 41, 331–341. doi: 10.1016/j.tibtech.2022.12.013 36710131 PMC9881846

[B53] Xiong H. He H. Chang Y. Miao B. Liu Z. Wang Q. . (2025). Multiple roles of NAC transcription factors in plant development and stress responses 67, 510–538. 10.1111/jipb.13854 39950532

[B54] Xu W. Zhu W. Shen J. Kuai M. Zhang Y. Huang W. . (2023). Stepwise rapid electrolytic synthesis of graphene oxide for efficient adsorption of organic pollutants. Nanoscale 15, 5919–5926. doi: 10.1039/d2nr06617c 36876907

[B55] Yan Y. Zhu X. Yu Y. Li C. Zhang Z. Wang F. (2022). Nanotechnology strategies for plant genetic engineering. Advanced Materials (Deerfield Beach Fla) 34, e2106945. doi: 10.1002/adma.202106945 34699644

[B56] Yang X. Zhang X. Liu Z. Ma Y. Huang Y. Chen Y. J. J. O. P. C. C. (2008). High-efficiency loading and controlled release of doxorubicin hydrochloride on graphene oxide. J. Phys. Chem. C 112, 17554–17558. doi: 10.1021/jp806751k

[B57] Zaidi S. S. Mansoor S. (2017). Viral vectors for plant genome engineering. Front. Plant Sci. 8, 539. doi: 10.3389/fpls.2017.00539 28443125 PMC5386974

[B58] Zhang X. Cao H. Zhao J. Wang H. Xing B. Chen Z. . (2021). Graphene oxide exhibited positive effects on the growth of Aloe vera L. Physiol. Mol. Biol. Plants: An. Int. J. Funct. Plant Biol. 27, 815–824. doi: 10.1007/s12298-021-00979-3 33967464 PMC8055783

[B59] Zhao G. Li J. Ren X. Chen C. Wang X. (2011). Few-layered graphene oxide nanosheets as superior sorbents for heavy metal ion pollution management. Environ. Sci. Technol. 45, 10454–10462. doi: 10.1021/es203439v 22070750

[B60] Zhao X. Liu P. J. R. A. (2014). Biocompatible graphene oxide as a folate receptor-targeting drug delivery system for the controlled release of anti-cancer drugs. RSC Adv. 4, 24232–24239. doi: 10.1039/c4ra02466d

