# Supplementary material for: High aspect ratio graphene oxide: a highly efficient plasmid DNA deliverer for plant seed
Source: Front Plant Sci. 2026 Jun 11;17:1855385. doi: 10.3389/fpls.2026.1855385 (PMC13294384; doi:10.3389/fpls.2026.1855385)
Supplement: Supplementary Figure 1 — Inverted fluorescence microscopy images of the leaf sections of spring wheat Zhongkemai 138 (ZKM138). Plants do not inherently emit fluorescence. The scale bar represents 50 µm. [file Image1.pdf]

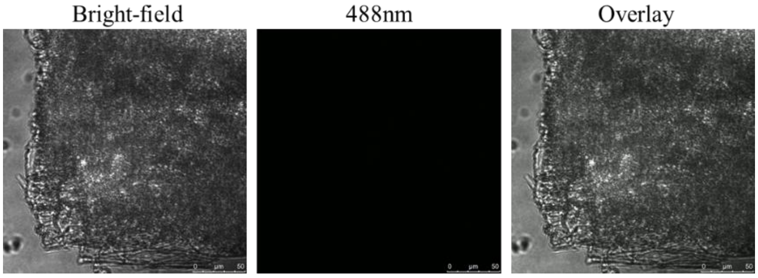

SUPPLEMENTARY FIGURE 1

Inverted fluorescence microscopy images of the leaf sections of spring wheat Zhongkemai 138 (ZKM138). Plants do not inherently emit fluorescence. The scale bar represents 50 μm.

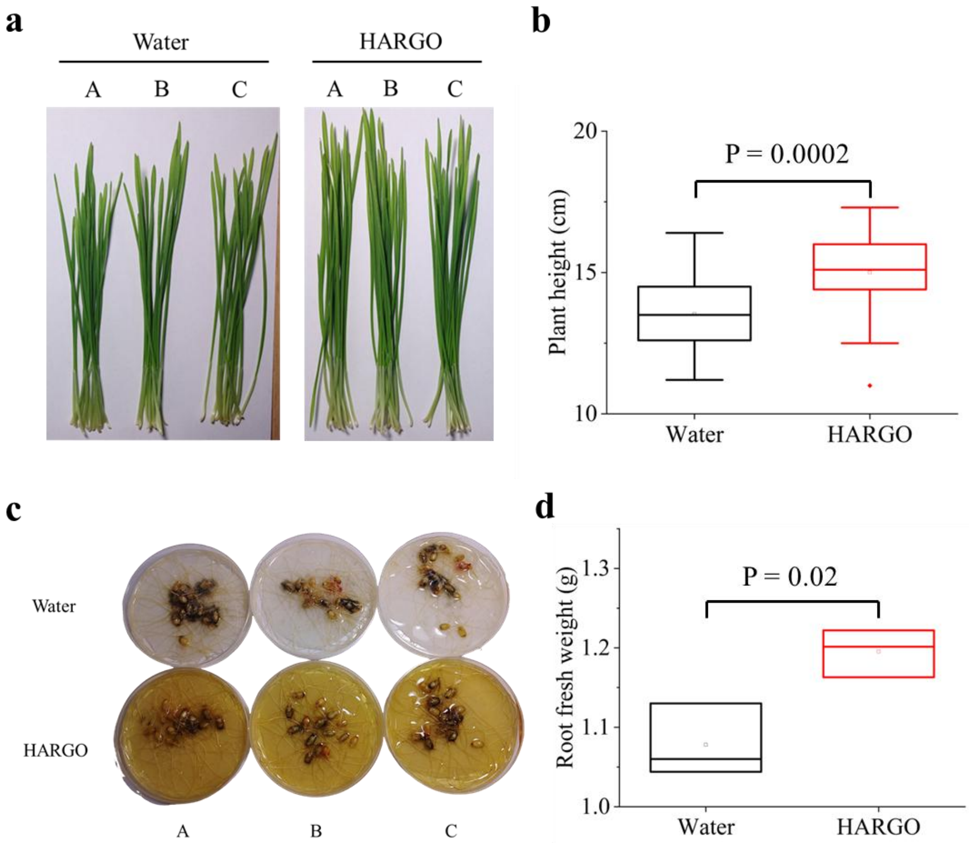

SUPPLEMENTARY FIGURE 2

Effect of high aspect ratio graphene oxide (HARGO) on wheat seedlings. **(a, b)**, Randomly select 15 seeds of spring wheat variety Zhongkemai 138 (ZKM138) and place them in a culture dish for germination. Digital photo of wheat seedlings 14 days after emergence **(a)** and their height statistics **(b)**. Among them, the number of seedlings in both groups is 45. **(c, d)**, Digital photo of roots remaining in the culture medium after harvesting wheat seedlings **(c)** and statistics of root fresh weight **(d)**.

a．

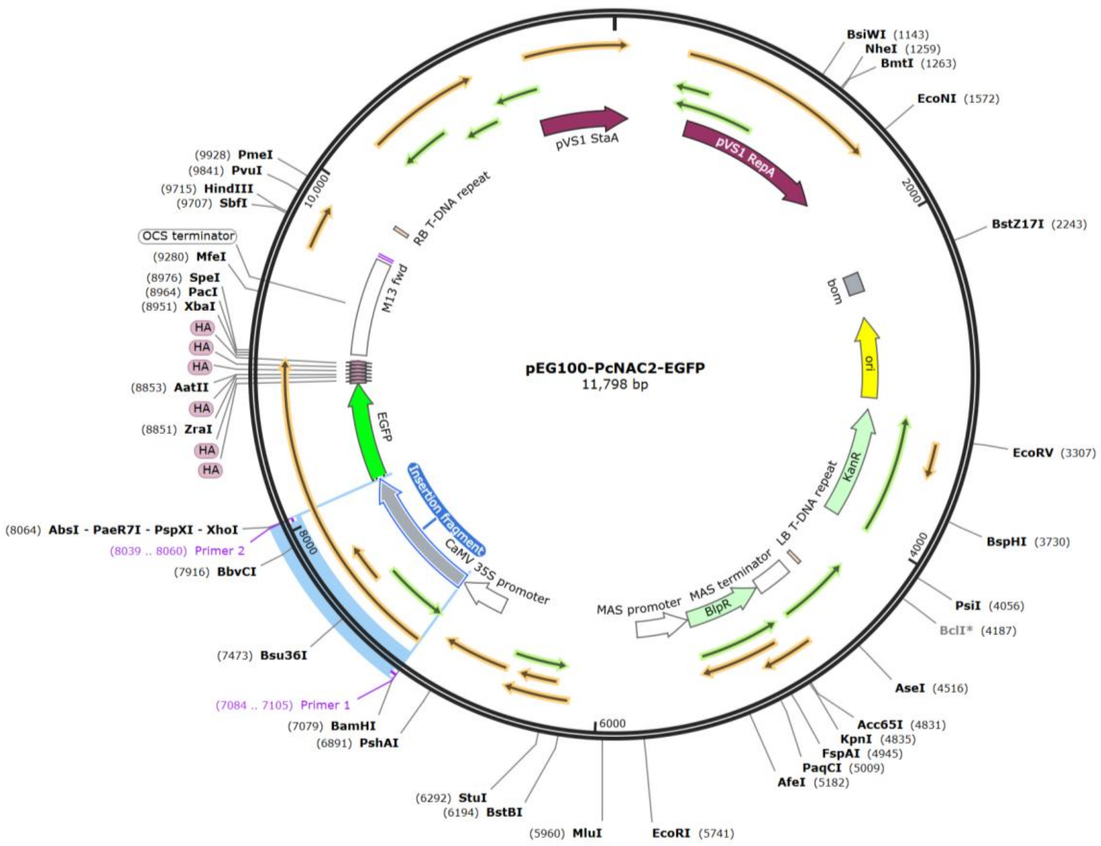

b. PcNAC2-EGFP Sequence

ATGGGGATGGCCGTGCGCAGGAGGGAGCGGGACGCGGAGGCGGACCTGAACCTGCC

GCCGGGCTTCCGGTTCCACCCGACGGACGACGAGCTCGTGGAGCACTACCTGTGCCG

CAAGGCCGCCGGGCAGCGCCTGCCGGTGCCCATCATCGCCGAGGTCGACCTCTACCGC

TTCGACCCCTGGGCGCTCCCCGACCGCGCCCTCTTCGGCACCCGCGAGTGGTACTTCT

TCACCCCGCGCGACCGCAAGTACCCCAACGGCTCCCGCCCCAACCGCGCCGCCGGCA

ACGGGTACTGGAAGGCCACCGGCGCCGACAAGCCCGTCGCGCCCCGCGGCGGGAAA

ACCATGGGGATCAAGAAGGCGCTCGTGTTCTACGCCGGGAAGGCGCCTAAGGGGGTG

AAGACGGATTGGATCATGCATGAGTATAGGCTCGCCGATGCTGGGCGGGCCAGCGCCG

TCGCCAAGAAAGGCTCACTCAGGTTGGACGACTGGGTTCTGTGCCGGCTGTACAACA

AGAAGAACGAGTGGGAGAAGATGCAGCAGGGGCAGCAGGGCGATCAGAAGGAGCCC

AAGGCGGAGGAGACGACGACGGCGTCGGACATGGTCACCTCGCAGTCCCACTCGCAC

TCGTGGGGCGAGGCGCGCACGCCGGAGTCGGAGATCGTGGACAACGACCCCTCGTCG

ATGATGTTACAGCAGGCGGCGGCCGCGGCGGGGTTCCAGAGCCCCGCGGCGCACCAG
GAGATGTTGGCCACGCTGATGGTGCCCAAGAAGGAGGCGGCGGACGAGGCTGGGAAC
GGGGCCGGTGGCAGGAACGACCTGTTCGTGGACCTCAGCTACGACGACATCCAGAGC
ATGTACAGCGGCCTCGACGTGATGCCGCCCGGGGACGACCTGCTCTACTCGTCCCTCT
TCGCCTCGCCCAAGCTCCGCGGGAACCAGAACGGCTCCGGCGGCATGCCGGCTCCCTT
CTTGAACCCTCGAGGGATGAGCAAGGGCGAGGAGCTGTTCACCGGGGTGGTGCCCAT
CCTGGTCGAGCTGGACGGCGACGTAAACGGCCACAAGTTCAGCGTGTCCGGCGAGGG
CGAGGGCGATGCCACCTACGGCAAGCTGACCCTGAAGTTCATCTGCACCACCGGCAA
GCTGCCCGTGCCCTGGCCCACCCTCGTGACCACCCTGACCTACGGCGTGCAGTGCTTC
AGCCGCTACCCCGACCACATGAAGCAGCACGACTTCTTCAAGTCCGCCATGCCCGAAG
GCTACGTCCAGGAGCGCACCATCTTCTTCAAGGACGACGGCAACTACAAGACCCGCG
CCGAGGTGAAGTTCGAGGGCGACACCCTGGTGAACCGCATCGAGCTGAAGGGCATCG
ACTTCAAGGAGGACGGCAACATCCTGGGGCACAAGCTGGAGTACAACTACAACAGCC
ACAACGTCTATATCATGGCCGACAAGCAGAAGAACGGCATCAAGGTGAACTTCAAGAT
CCGCCACAACATCGAGGACGGCAGCGTGCAGCTCGCCGACCACTACCAGCAGAACAC
CCCCATCGGCGACGGCCCCGTGCTGCTGCCCGACAACCACTACCTGAGCACCCAGTCC
GCCCTGAGCAAAGACCCCAACGAGAAGCGCGATCACATGGTCCTGCTGGAGTTCGTG
ACCGCCGCCGGGATCACTCTCGGCATGGACGAGCTGTACAAGTATCCTTACGATGTTCC
TGATTATGCATACCCATACGATGTACCAGACTACGCTTATCCATACGACGTCCCAGATTA
TGCCTATCCCTATGACGTGCCTGATTACGCATATCCGTATGATGTGCCTGACTATGCTTAC
CCATATGATGTTCCCGATTACGCTTAA

SUPPLEMENTARY FIGURE 3

The gene information of pEG100-PcNAC2-EGFP. **(a)**, Plasmid map of pEG100-PcNAC2-EGFP. **(b)**, The sequence of PcNAC2-EGFP. The sequence with a yellow background is the cDNA of PcNAC2 gene, the blue background represents a restriction site (XhoI), the green background shows EGFP, and the gray background indicates 6×HA.

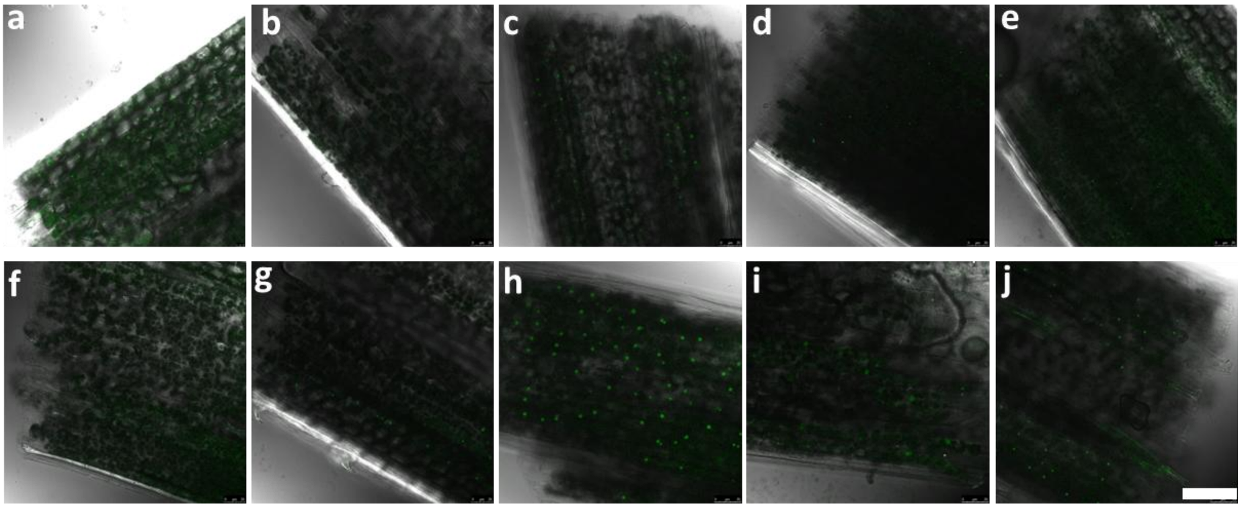

SUPPLEMENTARY FIGURE 4

Laser confocal microscopy images of ten 21-day-old Poa crymophila Keng seedlings. **(a–j)**, Confocal fluorescence microscopy images of seedlings germinated from ten independent Poa crymophila Keng seeds following the treatment with HARGO and plasmid solution. As shown in **(b)**, one seedling exhibited no detectable fluorescence; thus, the transfection efficiency was 90%. The excitation wavelength is 488 nm. The scale bar length is 50 μm.

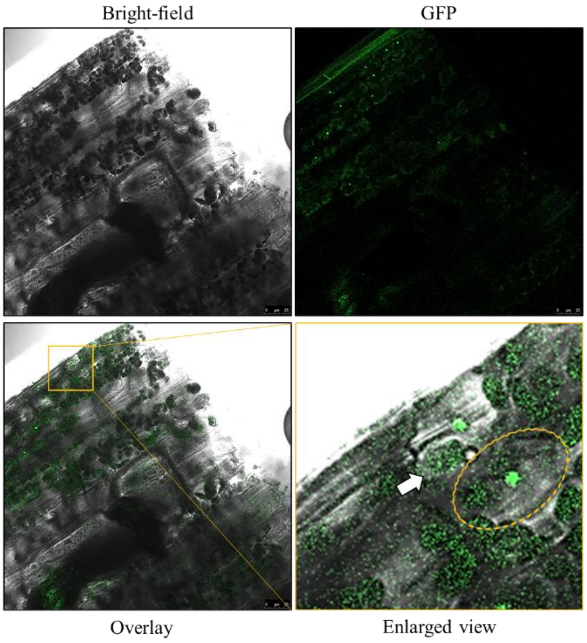

SUPPLEMENTARY FIGURE 5

Laser confocal microscopy images of the GFP-positive leaf tips. The yellow-framed image shows a magnified view of the region circled in the Overlay panel. A single cell is outlined by the yellow dashed box, and the green signal marked by the white arrow is likely derived from chloroplast autofluorescence. The scale bar represents 25 µm.
